# Artificial intelligence‐based assessment of leg axis parameters shows excellent agreement with human raters: A systematic review and meta‐analysis

**DOI:** 10.1002/ksa.12362

**Published:** 2024-07-21

**Authors:** Mikhail Salzmann, Hakam Hassan Tarek, Robert Prill, Roland Becker, Andreas G. Schreyer, Robert Hable, Marko Ostojic, Nikolai Ramadanov

**Affiliations:** ^1^ Center of Orthopaedics and Traumatology, Brandenburg Medical School University Hospital Brandenburg an der Havel Brandenburg an der Havel Germany; ^2^ Faculty of Health Science Brandenburg, Brandenburg Medical School Theodor Fontane Brandenburg an der Havel Germany; ^3^ Institute for Diagnostic and Interventional Radiology, Brandenburg Medical School Theodor Fontane Brandenburg an der Havel Germany; ^4^ Faculty of Applied Computer Science, Deggendorf Institute of Technology Deggendorf Germany; ^5^ Department of Orthopedics University Hospital Mostar Mostar Bosnia and Herzegovina

**Keywords:** AI, alignment measurement, artificial intelligence, automated measurement, deep learning, leg axis

## Abstract

**Purpose:**

The aim of this study was to conduct a systematic review and meta‐analysis on the reliability and applicability of artificial intelligence (AI)‐based analysis of leg axis parameters. We hypothesized that AI‐based leg axis measurements would be less time‐consuming and as accurate as those performed by human raters.

**Methods:**

The study protocol was registered with the International Prospective Register of Systematic Reviews (PROSPERO). PubMed, Epistemonikos, and Web of Science were searched up to 24 February 2024, using a BOOLEAN search strategy. Titles and abstracts of identified records were screened through a stepwise process. Data extraction and quality assessment of the included papers were followed by a frequentist meta‐analysis employing a common effect/random effects model with inverse variance and the Sidik–Jonkman heterogeneity estimator.

**Results:**

A total of 13 studies encompassing 3192 patients were included in this meta‐analysis. All studies compared AI‐based leg axis measurements on long‐leg radiographs (LLR) with those performed by human raters. The parameters hip knee ankle angle (HKA), mechanical lateral distal femoral angle (mLDFA), mechanical medial proximal tibial angle (mMPTA), and joint‐line convergence angle (JLCA) showed excellent agreement between AI and human raters. The AI system was approximately 3 min faster in reading standing long‐leg anteroposterior radiographs (LLRs) compared with human raters.

**Conclusion:**

AI‐based assessment of leg axis parameters is an efficient, accurate, and time‐saving procedure. The quality of AI‐based assessment of the investigated parameters does not appear to be affected by the presence of implants or pathological conditions.

**Level of Evidence:**

Level I.

**Abbreviations**
AI: artificial intelligence
AMA: anatomic‐mechanical angle
CI: confidence interval
CNN: convolutional neuronal network
DFO: distal femoral osteotomy
HKA: hip knee ankle angle
HTO: high tibial osteotomy
ICC: interclass correlation coefficient
JLCA: joint‐line convergence angle
LLR: standing long‐leg anteroposterior radiograph
mLDFA: mechanical lateral distal femoral angle
mMPTA: mechanical medial proximal tibial angle
PRISMA: preferred reporting items for systematic reviews and meta‐analysis
RCT: randomized controlled trial
RoB: risk of bias
ROBINS‐I: risk of bias in non‐randomized studies of interventions
TKA: total knee arthroplasty

## INTRODUCTION

Analysis of the leg axis and configuration is crucial for diagnosing leg deformities and planning surgical interventions, particularly corrective osteotomy or arthroplasty [11, 21, 22]. These parameters are also vital for postoperative quality control [17, 24]. Typically, they are measured using standing long‐leg anteroposterior radiographs (LLR) [33]. Consequently, significant efforts have been made to classify leg configurations and improve the predictability of surgical procedures [6, 15]. Currently, these measurements are performed by radiologists or orthopaedic surgeons, either manually drawing lines and angles on digital radiographs or using specialized software that requires manual referencing of bony structures. Both methods are time‐consuming and lack reproducibility [16, 29]. Additionally, measurement accuracy has been reported to be highly dependent on the rater's experience [38].

In recent years, efforts in medicine have focused on enabling artificial intelligence (AI) to establish accurate diagnoses and suggest appropriate therapies [8]. In orthopaedics and traumatology, AI aids in detecting conditions such as fractures, dislocations, bone lesions, and axis deviations [12]. The American College of Radiology Data Science Institute has recently identified the measurement of leg length discrepancies in radiographs as a suitable application for AI to improve medical care. As with all new medical procedures, it is essential to prove that AI is a valid, reliable, effective, and useful tool for clinical practice. In this context, there has been a significant increase in medical publications on the role of AI in medicine [13]. However, these publications often focus on self‐developed AI applications and lack comparability. Thus, scientific knowledge on the practical application of AI for determining leg axis parameters remains insufficiently investigated. Above all, there is still no systematic review and meta‐analysis to provide concrete conclusions.

The aim of this study was to conduct a systematic review and meta‐analysis of all relevant studies on the reliability and applicability of AI in determining radiological leg axis parameters and to formulate initial summarizing conclusions. We hypothesized that AI‐based leg axis measurement would be less time‐consuming and as accurate as measurements performed by human raters.

## METHODS

The study protocol was registered in the International Prospective Register of Systematic Reviews (PROSPERO) on 12 February 2024 [CRD42024508249]. The updated version of the Preferred Reporting Items for Systematic Reviews and Meta‐Analyses (PRISMA) guidelines was strictly followed for reporting [20]. Author Guidelines for Systematic Reviews and Meta‐Analysis were adhered to throughout the process [25]. A PRISMA checklist was completed and made available as supplementary material in the published version of the study [26]. PubMed, Epistemonikos, and Web of Science were searched up to 24 February 2024, using a BOOLEAN search strategy. No restrictions on language or year of publication were applied. The search terms were adapted to the specific search modalities of each platform: (((Neuronal Networks) OR (Deep Learning) OR (AI) OR (Artificial Intelligence)) AND ((leg axis) OR (hip knee ankle angle) OR (long‐leg radiograph))).

### Screening and selection of studies

In a stepwise screening process, the titles and abstracts of the identified records were screened after duplicates have been removed independently by two experienced orthopaedic surgeons (M. S. and N. R.). Discrepancies were solved in discussion. The screened articles were then assessed for eligibility by full text analysis. The final decision on in‐ and exclusion for each study was made through consensus between two reviewers after scientific discussion (M. S. and N. R.), Kappa coefficient (*κ*) was calculated concerning inclusion consistency.

In a stepwise screening process, the titles and abstracts of the identified records were independently screened by two experienced orthopaedic surgeons (M. S. and N. R.) after duplicates were removed. Discrepancies were resolved through discussion. The screened articles were then assessed for eligibility through full‐text analysis. The final decision on inclusion and exclusion for each study was made by consensus between the two reviewers after scientific discussion (M. S. and N. R.). The Kappa coefficient (*κ*) was calculated to assess inclusion consistency.

### Inclusion and exclusion criteria

All types of studies with primary data, including randomized controlled trials (RCTs), controlled trials, cohort studies, and case‐control studies involving human participants with any indication for whole leg radiographs and AI and clinical assessment of leg axis parameters were included. The primary outcome measure was defined as the accuracy of AI‐based leg axis assessment determined by comparison with human assessment. The parameters extracted in particular were the hip knee ankle angle (HKA), mechanical lateral distal femoral angle (mLDFA), mechanical medial proximal tibial angle (mMPTA), and joint‐line convergence angle (JLCA) The time needed for these measurements and the differences between AI and rater‐based measurements were defined and extracted as separate outcome parameters. Case reports, case series, and animal studies were excluded.

### Data extraction

Two reviewers (M. S. and H. T. H.) independently extracted all data on study characteristics, methods, parameters for subsequent quality assessment, participant characteristics, accuracy of AI‐based leg axis assessment, and relevant additional information. Disagreements were resolved by consensus. The raw data extraction set is available in the Supporting Information of the published version (Supporting Information: S1), as well as a tabular summary of the studies included (Supporting Information: S2 and S3). The corresponding authors of the included studies were contacted to obtain missing primary data.

### Quality assessment of the included studies

Two reviewers (N. R. and H. T. H.) independently assessed the quality of the included studies. Risk of bias assessment was performed using the JBI Critical Appraisal tool [26]. Additionally, publication bias was assessed using Begg's and Egger's tests, presented in a funnel plot.

### Measures of treatment effect

A frequentist meta‐analysis was performed using a common effect/random effects model with inverse variance and the Sidik–Jonkman heterogeneity estimator. The intraclass correlation coefficient (ICC) and mean difference (MD) with 95% confidence intervals (CIs) were calculated using Fisher's *z*‐transformation. Study heterogeneity was assessed using the Higgins test *I*² (low heterogeneity <25%, moderate heterogeneity: 25%–75%, and high heterogeneity >75%) [5]. As some outcome parameters indicated high heterogeneity, a random effects model was used to present the results.

## RESULTS

**Figure 1:**
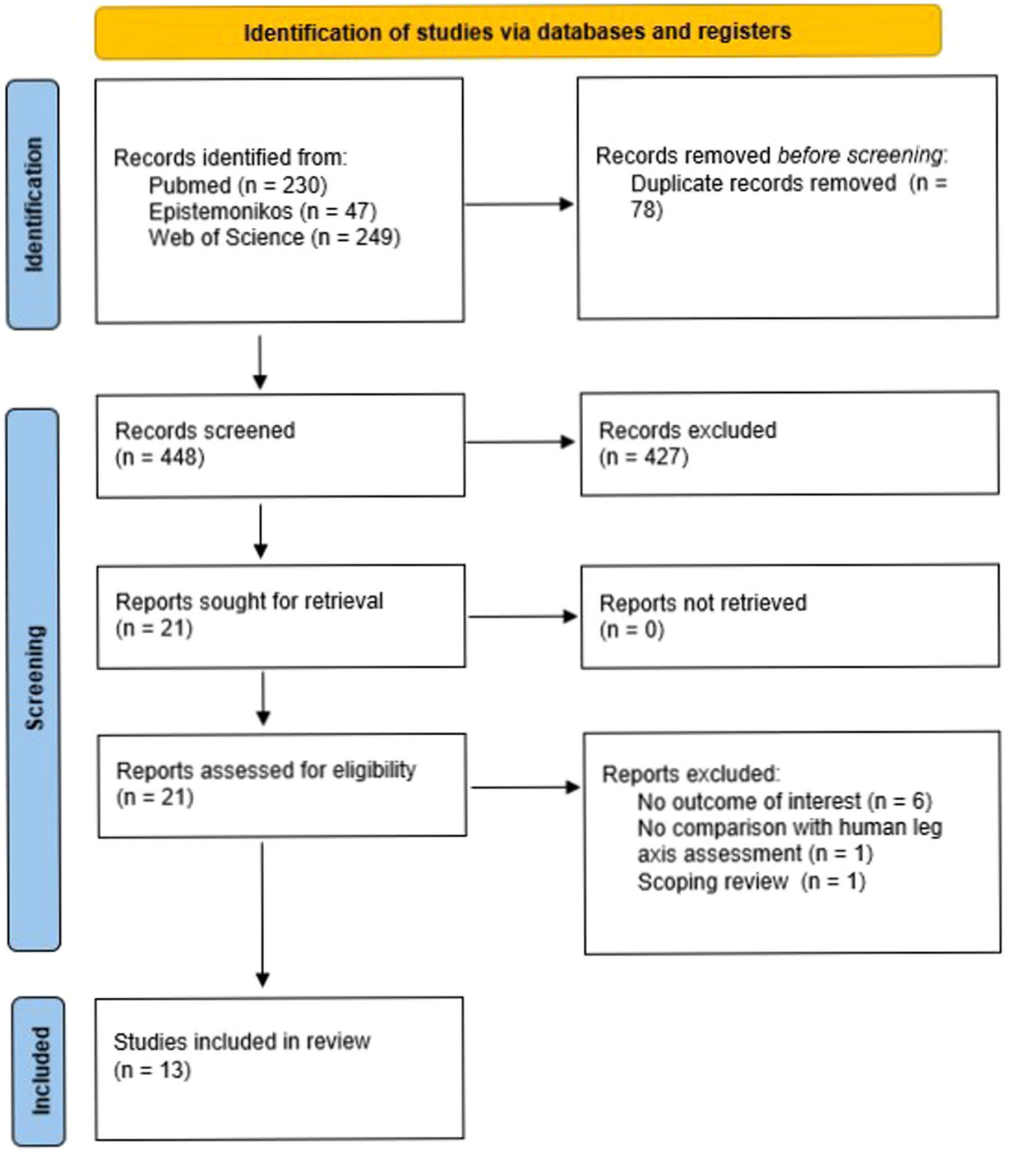
PRISMA flow diagram of the search results and selection according to our inclusion criteria.

After excluding 78 duplicates, the initial literature search yielded 448 records. After title and abstract screening, 21 records remained for further consideration, with high inter‐reviewer agreement (*κ* = 0.95). Of these 21 studies [1, 2, 3, 4, 7, 9, 10, 13, 18, 19, 23, 27, 28, 30, 31, 32, 34, 35, 36, 37, 39], eight were excluded [*κ* = 1.0] for the following reasons: six studies did not report an outcome of interest [2, 9, 27, 28, 36, 39], one study did not compare AI results with human leg axis assessment [7], and one study was a scoping review with a different topic [4]. Finally, 13 studies were included in the systematic review [1, 3, 10, 13, 18, 19, 23, 30, 31, 32, 34, 35, 37]. The entire search process is presented in a PRISMA flow chart (Figure 1).

### Characteristics of the studies included

The 13 included studies were published between 2020 and 2023 and included a total of 3192 patients who had their leg axis assessed. The mean age of the patients was 41 years (range: 11–66). In five of the studies, a vendor‐provided deep‐learning‐based software (IB Lab LAMA, IB Lab GmbH) was used for leg axis assessment [1, 18, 31, 32, 35]. The other eight studies used self‐developed and self‐validated AI solutions [3, 10, 13, 19, 23, 30, 34, 37]. Among the included studies, five included patients without any implants [1, 19, 23, 32, 34], four included patients without any implants, as well as those after knee replacement [3, 10, 13, 30], two included patients after osteotomy [18, 35], and one included only patients after total knee replacement [31]. One study reported leg axis assessment in children [37]. More details about the included studies are presented in Table 1.

**Table 1:** Main characteristics of the studies included in meta‐analysis.

Primary study	Year of publication	Origin of publication	Patients, *n*	AI‐type	LLR characteristics	Human raters, *n*	Outcome
Archer et al. [1]	2023	USA, Austria, UK	132	LAMA™ (IB Lab GmbH, Vienna, Austria)	no implants	2	HKA; mLDFA; mMPTA; JLCA; time
Erne et al. [3]	2022	Germany, USA	95	self‐developed	before (n = 95) and after TKA (n = 105)	2	HKA; mLDFA; mMPTA; JLCA
Jo et al. [10]	2023	South Korea	305	self‐developed	with or without implants, only right side	2	HKA; mLDFA; mMPTA; JLCA
Larson et al. [13]	2021	USA	220	self‐developed	with or without implants	3	HKA
Mitterer et al. [18]	2023	Austria	110	LAMA™ (IB Lab GmbH, Vienna, Austria)	after HTO (n = 55) and after DFO (n = 55)	3	HKA; mLDFA; mMPTA; JLCA; time
Moon et al. [19]	2023	South Korea	200	self‐developed	no implants	1	HKA; mLDFA; mMPTA; JLCA
Pei et al. [23]	2020	China	120	self‐developed	no implants	3	HKA
Schock et al. [30]	2021	Germany	212	self‐developed	with or without implants	2	HKA; time
Schwarz et al. [31]	2022	Austria	174	self‐developed	after T28,KA only		HKA
Simon et al. [32]	2021	Austria	295	LAMA™ (IB Lab GmbH, Vienna, Austria)	no Implants	2	HKA; mLDFA; mMPTA; JLCA; time
Steele et al. [34]	2023	USA	134	self‐developed	no implants	1	HKA; mLDFA; mMPTA; JLCA
Stotter et al. [35]	2023	Austria, Germany	181	LAMA™ (IB Lab GmbH, Vienna, Austria)	before and after HTO	3	HKA; mLDFA; mMPTA; JLCA
Tsai et al. [37]	2021	USA	1014	self‐developed	children only	1	HKA

Abbreviations: AI, artificial intelligence; HKA, hip knee ankle angle; JLCA, joint‐line convergence angle; LLR, standing long‐leg anteroposterior radiograph; mLDFA, mechanical lateral distal femoral angle; mMPTA, mechanical medial proximal tibial angle.

### Quality assessment

The results of the risk of bias assessment using the revised JBI Critical Appraisal Tool are presented in Table 2. No relevant publication bias was found. The publication bias calculated using Begg's and Egger's tests is shown in Table 3.

**Table 2:** Risk of bias assessment with the revised JBI Critical Appraisal Tool.

JBI cohort studies	Groups comparable	Exposure measured in both groups	Valid method for Identification	Confounding factors identified	Strategy for confounders	Groups free of outcome at start	Outcomes measured valid	Follow up sufficient	Follow up complete/strategies for incomplete	Statistics
Archer et al. [1]	‐	+	+	X	X	‐	+	X	X	+
Erne et al. [3]	‐	+	+	+	X	+	+	X	X	+
Jo et al. [10]	‐	+	‐	+	X	‐	+	X	X	+
Larson et al. [13]	‐	+	+	‐	X	+	+	X	X	+
Mitterer et al. [18]	‐	‐	+	+	‐	X	+	X	X	+
Moon et al. [19]	‐	‐	+	X	X	+	+	X	X	+
Pei et al. [23]	‐	‐	+	‐	+	‐	+	X	X	+
Schock et al. [30]	‐	‐	+	+	X	+	+	X	X	+
Schwarz et al. [31]	‐	+	+	‐	‐	X	+	X	X	+
Simon et al. [32]	‐	+	+	‐	X	+	+	X		
Steele et al. [34]	‐	‐	+	+	X	‐	+	X	X	+
Stotter et al. [35]	‐	+	+	+	X	+	+	X	‐	+
Tsai et al. [37]	‐	+	+	+	X	‐	+	X	X	+

Abbreviations: +, fulfilled; ‐ unclear; X, not fulfilled.

**Table 3:** Results of the meta‐analysis for all outcome parameters included.

	Number of primary studies	Number of patients	Treatment effect random	*p* Value random	Treatment effect common	*p* Value common	*I* ^2^	Tau2	Type	Egger bias	Egger *p* value
HKA interrater	5	823	0.99	<0.0001***	0.99	<0.0001***	0	0	ICC	0	0.7601
HKA AI versus rater	12	3011	0.99	<0.0001***	0.99	<0.0001***	0.99	0.4	ICC	6.94	0.3807
mLDFA Interrater	5	823	0.94	<0.0001***	0.95	<0.0001***	0.91	0.06	ICC	−10.19	0.1403
mLDFA AI versus rater	7	1271	0.95	<0.0001***	0.96	<0.0001***	0.97	0.19	ICC	−19.94	0.0081**
mMPTA interrater	5	823	0.95	<0.0001***	0.96	<0.0001***	0.96	0.16	ICC	−12.79	0.2697
mMPTA AI versus rater	7	1271	0.96	<0.0001***	0.97	<0.0001***	0.98	0.24	ICC	−15.74	0.187
JLCA Interrater	4	728	0.91	<0.0001***	0.93	<0.0001***	0.98	0.23	ICC	−15.59	0.4937
JLCA AI versus rater	6	1176	0.93	<0.0001***	0.94	<0.0001***	0.97	0.13	ICC	−7.9	0.5648
Time	5	1898	−178.01	0.0271*	−60.61	<0.0001***	1	32,445.36	ICC	−130.5	0.0495*

*Note*: * statistically significant, ** highly statistically significant, and *** very highly statistically significant.

Abbreviations: AI, artificial intelligence; HKA, hip knee ankle angle; ICC, interclass correlation coefficient; JLCA, joint‐line convergence angle; mLDFA, mechanical lateral distal femoral angle; mMPTA, mechanical medial proximal tibial angle.

### Meta‐analysis

#### HKA agreement between human raters

**Figure 2:**
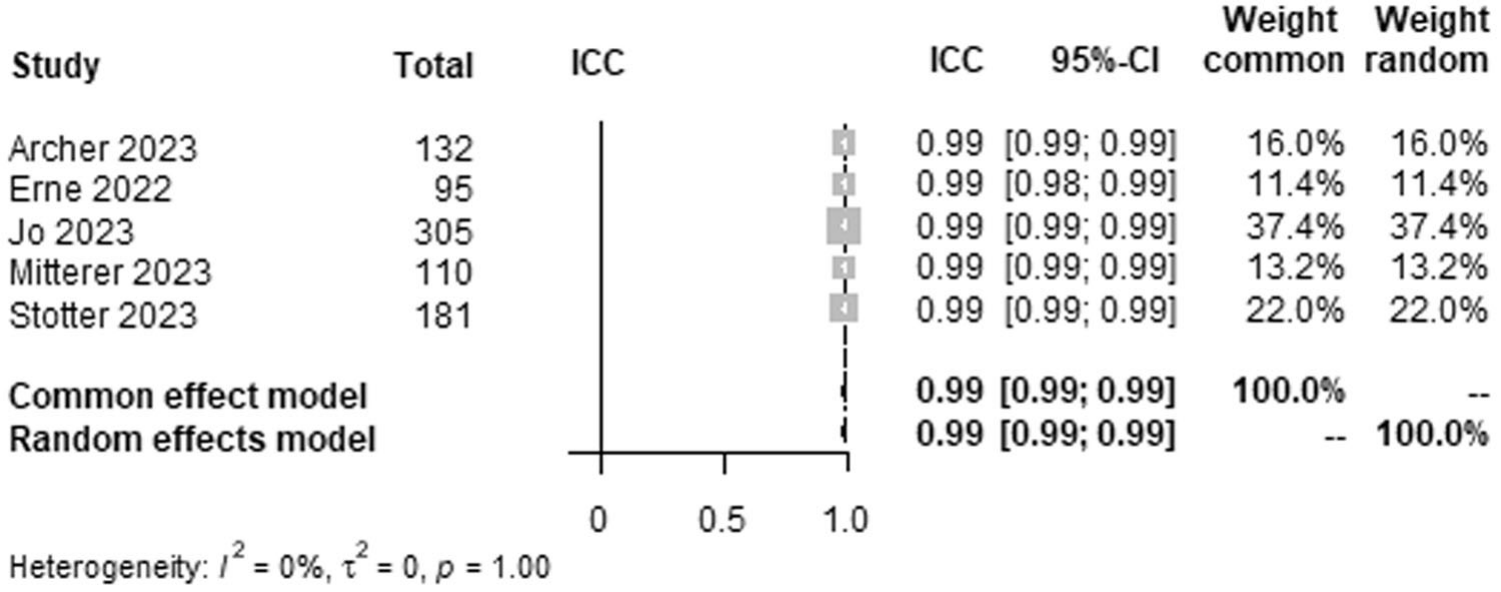
Forest plots of the pooled studies—HKA agreement between human raters. CI, confidence interval; HKA, hip knee ankle angle; ICC, interclass correlation coefficient.

To analyse interrater agreement for HKA on LLR, data from 823 patients from five studies were pooled (Figure 2, Table 3). ICC estimates between raters showed excellent agreement (ICC = 0.99, 95% CI 0.99–0.99; *I*² = 0%).

#### HKA agreement between AI and human rater

**Figure 3:**
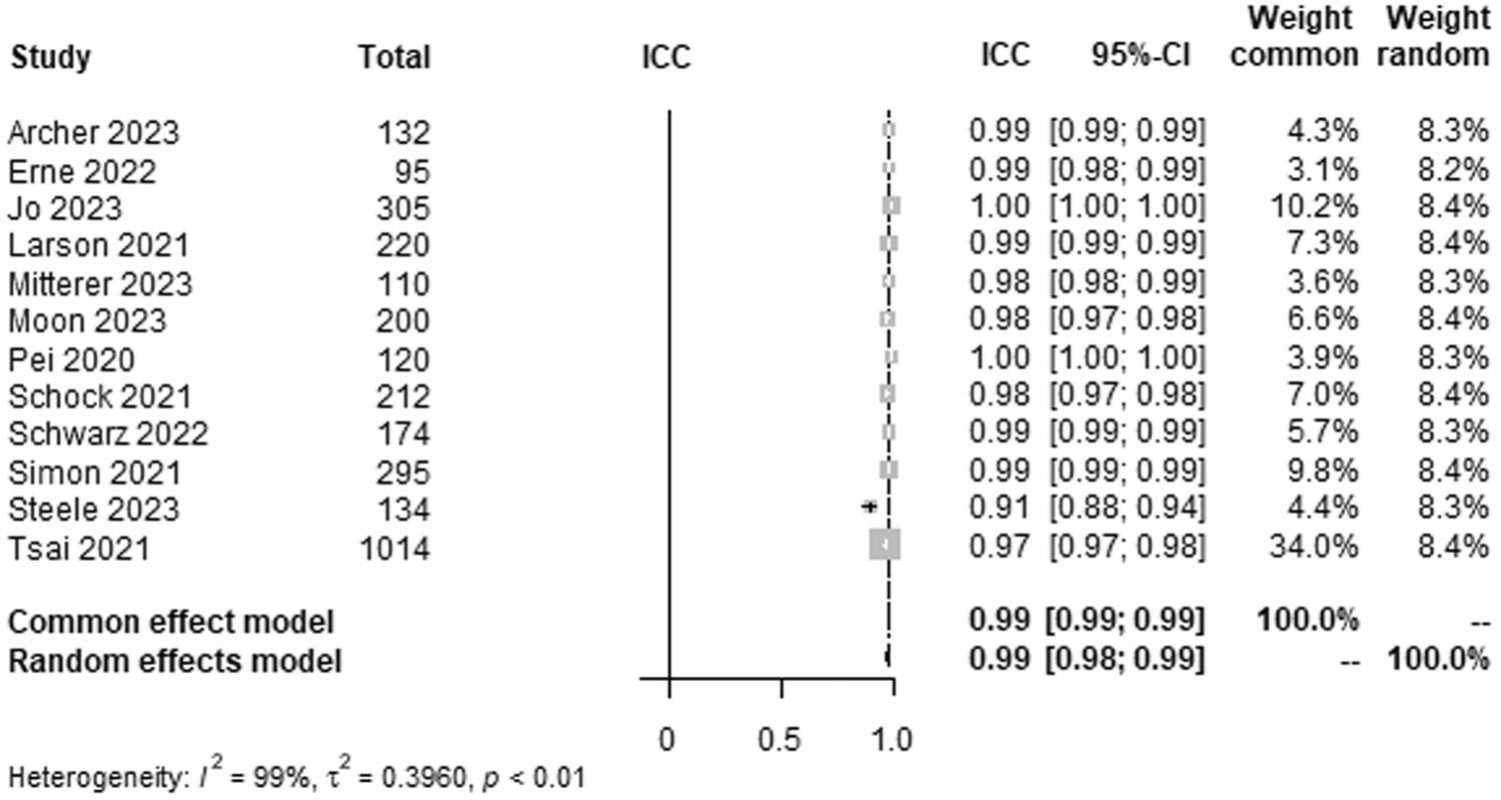
Forest plots of the pooled studies—HKA agreement between AI and human rater. AI, artificial intelligence; CI: confidence interval; HKA, hip knee ankle angle; ICC, interclass correlation coefficient.

To analyse the agreement between AI and rater for HKA on LLR, data from 3011 patients from 12 studies were pooled (Figure 3, Table 3). ICC estimates between AI and rater showed excellent agreement (ICC = 0.99, 95% CI 0.98–0.99; *I*² = 99%).

#### mLDFA agreement between human raters

**Figure 4:**
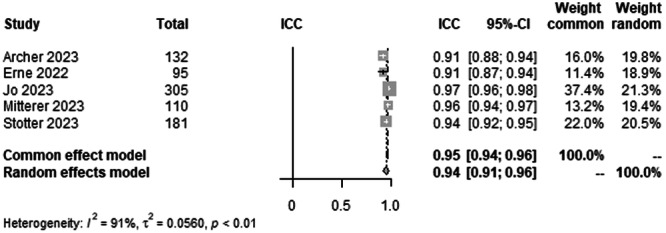
Forest plots of the pooled studies—mLDFA agreement between human raters. CI, confidence interval; ICC, interclass correlation coefficient; mLDFA, mechanical lateral distal femoral angle.

To analyse interrater agreement for mLDFA on LLR, data from 823 patients from five studies were pooled (Figure 4, Table 3). ICC estimates between raters showed excellent agreement (ICC = 0.94, 95% CI 0.91–0.96; *I*² = 91%).

#### mLDFA agreement between AI and human rater

**Figure 5:**
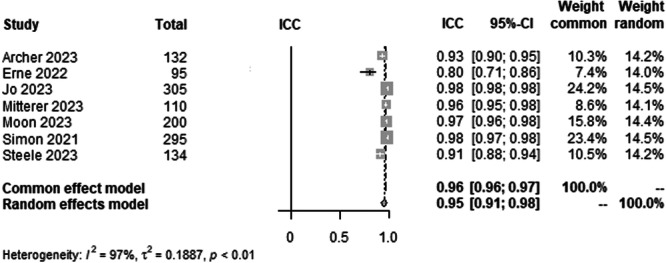
Forest plots of the pooled studies—mLDFA agreement between AI and human rater. AI, artificial intelligence; CI, confidence interval; ICC, interclass correlation coefficient; mLDFA, mechanical lateral distal femoral angle.

To analyse the agreement between AI and rater for mLDFA on LLR, data from 1271 patients from seven studies were pooled (Figure 5, Table 3). ICC estimates between AI and rater showed excellent agreement (ICC = 0.95, 95% CI 0.91–0.98; *I*² = 97%).

#### mMPTA agreement between human raters

**Figure 6:**
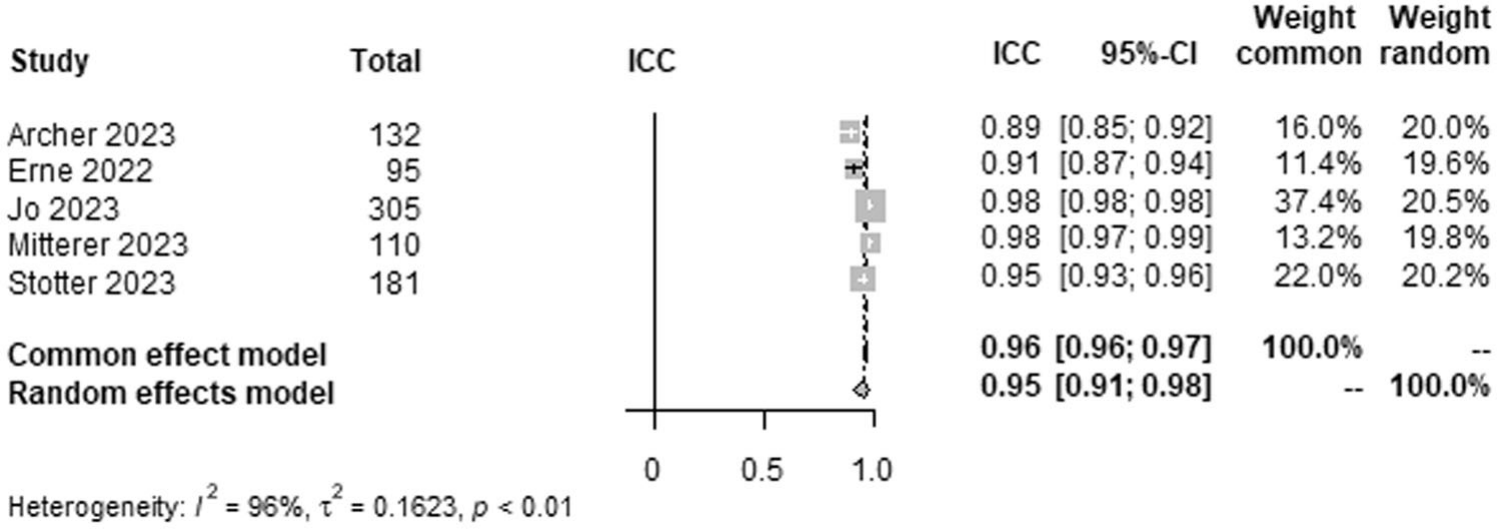
Forest plots of the pooled studies mMPTA agreement between human raters. CI, confidence interval; ICC, interclass correlation coefficient; mMPTA, mechanical medial proximal tibial angle.

To analyse interrater agreement for mMPTA on LLR, data from 823 patients from five studies were pooled (Figure 6, Table 3). ICC estimates between raters showed excellent agreement (ICC = 0.95, 95% CI 0.91–0.98; *I*² = 96%).

#### mMPTA agreement between AI and human rater

**Figure 7:**
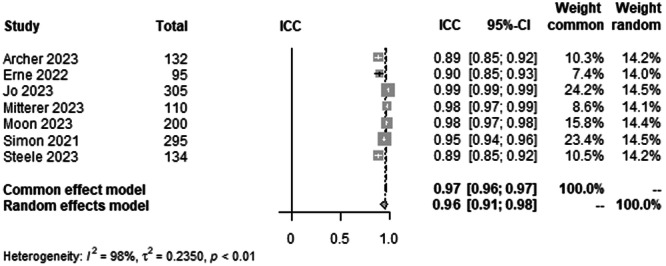
Forest plots of the pooled studies—mMPTA agreement between AI and human rater. AI, artificial intelligence; CI, confidence interval; ICC, interclass correlation coefficient; mMPTA, mechanical medial proximal tibial angle.

To analyse the agreement between AI and rater for mMPTA on LLR, data from 1271 patients from seven studies were pooled (Figure 7, Table 3). ICC estimates between AI and rater showed excellent agreement (ICC = 0.95, 95% CI 0.91–0.98; *I*² = 98%).

#### JLCA agreement between human raters

**Figure 8:**
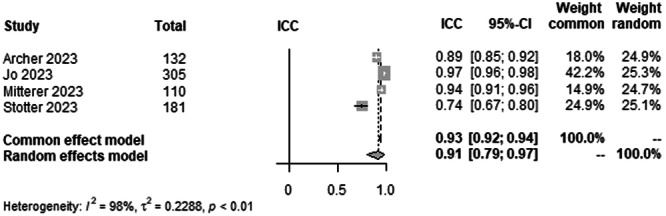
Forest plots of the pooled studies—JLCA agreement between human raters. CI, confidence interval; ICC, interclass correlation coefficient; JLCA, joint‐line convergence angle.

To analyse interrater agreement for JLCA on LLR, data from 728 patients from four studies were pooled (Figure 8, Table 3). ICC estimates between raters showed excellent agreement (ICC = 0.91, 95% CI 0.79–0.97; *I*² = 98%).

#### JLCA agreement between AI and human rater

**Figure 9:**
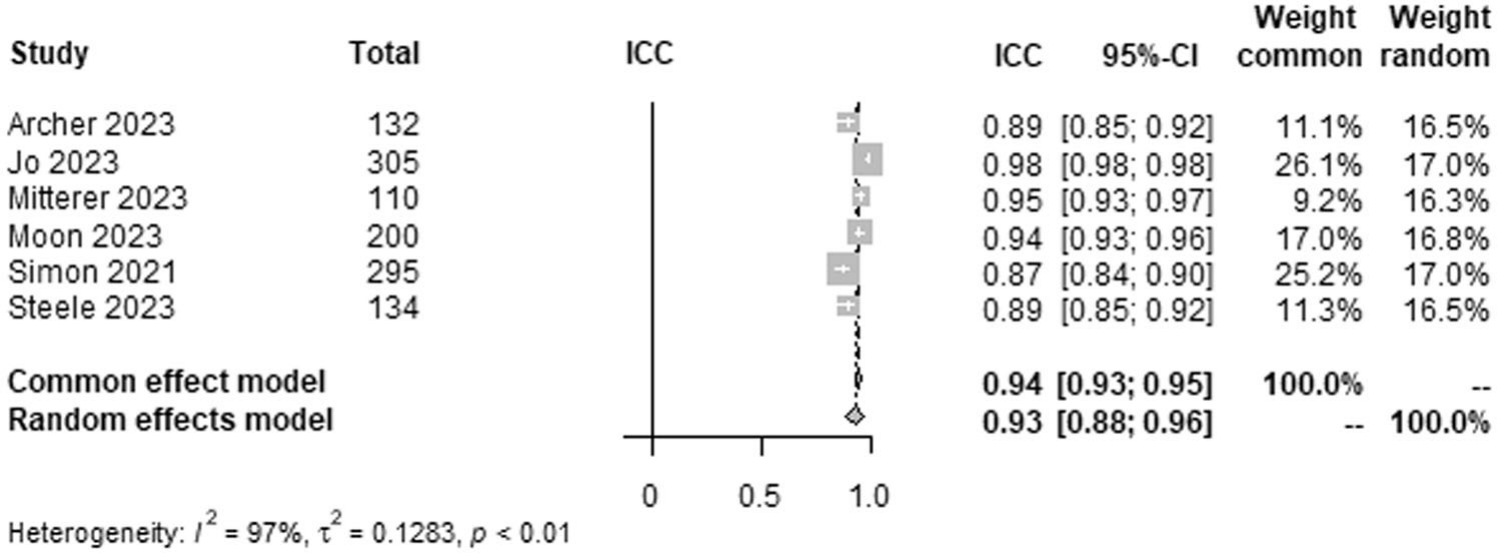
Forest plots of the pooled studies—JLCA agreement between AI and human rater. AI, artificial intelligence; CI, confidence interval; ICC, interclass correlation coefficient; JLCA: joint‐line convergence angle.

To analyse the agreement between AI and rater for JLCA on LLR, data from 1176 patients from six studies were pooled (Figure 9, Table 3). ICC estimates between AI and rater showed excellent agreement (ICC = 0.93, 95% CI 0.88–0.96; *I*² = 97%).

#### Time for assessing LLR

**Figure 10:**
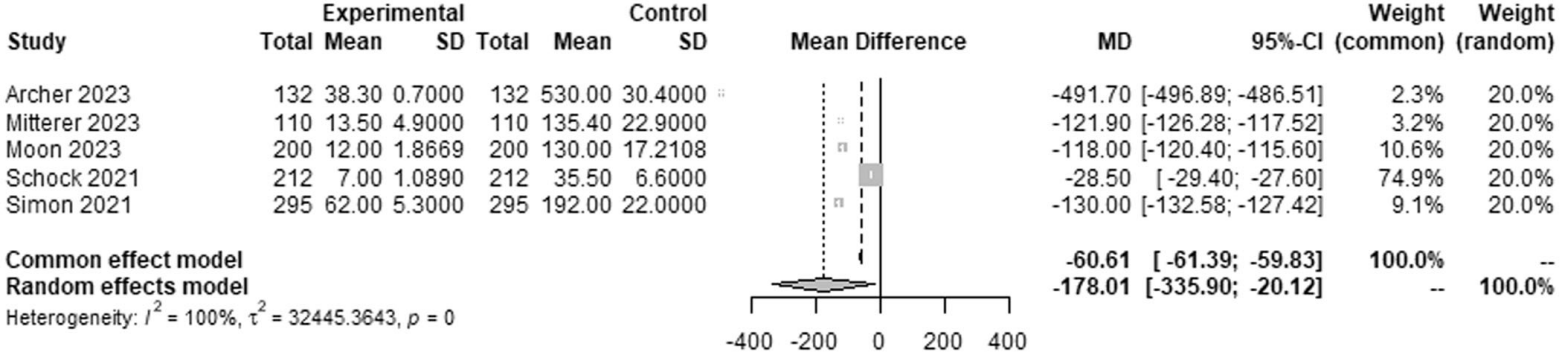
Forest plots of the pooled studies—time for assessing LLR. CI, confidence interval; LLR, standing long‐leg anteroposterior radiograph; MD, mean difference; SD, standard deviation.

To analyse the time to read the LLR of the AI compared with the rater, data from 1898 patients from five studies were pooled (Figure 10, Table 3). The time to read the LLR of the AI was 178 s faster than the time to read the LLR of the rater (MD = −178.01, 95% CI −335.90 to −20.12; *I*² = 100%).

## DISCUSSION

The most important finding of this study was that all leg axis parameters showed excellent agreement between AI and raters. The time to read the LLR of the AI was approximately 3 min faster compared with a human rater. For the HKA, ICC estimates between AI and raters showed excellent agreement (ICC = 0.99) with a CI from 0.98 to 0.99. The mLDFA and the mMPTA in a pool of 1271 patients from seven studies showed excellent ICC estimates between AI and raters (ICC = 0.95), but with a slightly lower CI varying from 0.91 to 0.98 when compared with the HKA ICC. The study by Erne et al. [3]. showed the lowest ICC values for the mLDFA (Figure 5) compared with the other studies [1, 10, 13, 18, 19, 23, 30, 31, 32, 34, 35, 37]. However, the preoperative patient cohort, which was included in this meta‐analysis, was the smallest in this group with only 95 patients, which could be a possible explanation. The ICC estimates between AI and raters for the JLCA were also excellent by definition but with lower ICC values (ICC = 0.93) and a higher range of variability than all other parameters. Here, the CI for interrater ICC of AI and raters was 0.79–0.97 and 0.88–0.96, respectively. This, however, was the smallest pool with 728 patients from four studies [1, 10, 18, 35]. A possible conclusion could be that higher absolute values of the measured angles show a higher interclass correlation than smaller angles, with the JLCA being the smallest angle measured [14]. The work by Stotter et al. [35] showed lower ICC values for interrater reliability, which further supports this assumption.

The studies included in this meta‐analysis showed certain limitations that should be considered when designing future studies. While some studies [1, 19, 23, 32, 34] tried to analyse the capabilities of AI‐based software for angle detection in LLRs with no known pathologies, others [37] explicitly limited the analysis to pathological conditions such as congenital malformations. Furthermore, some studies [3, 10, 13, 18, 30, 31] included radiographs with hardware (total knee arthroplasties, unicondylar knee arthroplasties, high tibial osteotomies, and osteosynthesis material), while other studies excluded these subsets from training or analysis [1, 19, 23, 32, 34]. Additionally, studies aiming to develop new AI software used different sizes of datasets [3, 10, 13, 19, 23, 30, 31, 34, 37]. In studies with smaller datasets, this might have led to the over‐ or underestimation of the results. Studies that aimed to quantify the accuracy of existing models can be contrasted with studies aiming to develop, validate, and then test the AI software at hand. While five authors used the vendor‐provided LAMA™ solution in their studies [1, 18, 31, 32, 35], other authors used self‐developed solutions [3, 10, 13, 19, 23, 30, 34, 37]. Furthermore, different AI models used different convolutional neuronal networks (CNNs) to complete a spectrum of tasks, while some others used a single CNN to complete the measurement of a single angle. In addition, several studies [3, 10, 13, 19, 23, 30, 31, 32, 37] established a ground truth with an expert reader and measured the AI model against it. In other studies [1, 18, 34, 35], manual reading was achieved by several readers with different levels of clinical expertise.

It is noteworthy that authors have reported a manual preselection process of images in their studies. Images that did not match radiological quality standards, radiographs of patients with severe obesity or images that failed to meet the quality criteria of the investigated AI algorithm have been excluded. The ability to recognize an image as unsuitable seems, therefore, to be a limitation of AI‐based leg axis assessment. Ideally, an AI algorithm should be able to detect unsuitable images by itself and inform the clinician about the necessity to repeat the radiograph. At the moment, analysis of huge datasets without any human preselection does not seem to be possible.

Pooled data from 1898 patients in five studies showed that AI was 178 s faster than human raters in LLR analysis. In some studies, the AI was up to 10 times faster than a human rater [1, 19, 23]. At the same time, AI was just as accurate. In this context, it should be noted that AI‐based application working speed is directly linked to computing power. More powerful computers will provide faster AI‐based measurements, making it possible to screen and measure large datasets in a shorter period of time [3]. Schock et al. reported that a single bilateral LLR analysis took 3 s on a dedicated workstation and 7 s on a consumer‐grade laptop [30]. In addition, the time and individual steps measured varied throughout the different studies. For example, Schock et al. measured the time needed for a human rater to perform bilateral measurements of the HKA and AMA, matching it against a self‐developed AI solution, whereas Mitterer et al. summarized all steps to compare them to the AI software's workflow and included the overall time for the manual measurements as well as uploading the image, annotating it, extracting, and saving the measurements while matching it against LAMA™ [18].

In the future, AI‐based leg axis assessment has the potential for enormous time savings in everyday practice without the loss of quality and accuracy. Being faster and, at the same time, just as accurate as a human professional will also have an economic impact on how human time resources in radiology and orthopaedic surgery are addressed. At the same time, potential risk factors such as false predictions must be taken seriously. Mitigating and preventing these risks will be an essential task in the future [40]. Currently, however, we feel that AI has to become much more ‘intelligent’ to master this task. Therefore, further research on AI‐based image assessment will be needed to validate and develop tools that will perform these tasks safely, accurately, and more independently of human quality control.

The limitations of this study are as follows: (i) The patient cohorts of the included studies were heterogeneous, combining patients with total or unicondylar knee arthroplasty, tibial osteotomy, osteosynthesis material, or no surgery. (ii) Similarly, the AI applications used were heterogeneous (LAMA™ or self‐developed applications), and the human raters had different professional qualifications. (iii) Most of the outcome parameters analysed showed a high degree of heterogeneity.

## CONCLUSION

In conclusion, our work shows that AI‐based assessment of leg axis parameters is an efficient, accurate, and time‐saving procedure. The quality of AI‐based assessment of the parameters investigated does not seem to be affected by the presence of implants or pathological conditions. At present, AI does not appear to be capable of differentiating between suitable and unsuitable images for its measurements and requires human quality control.

## AUTHOR CONTRIBUTIONS

All authors contributed to the study conception and design. Material preparation and data collection were performed by Mikhail Salzmann, Nikolai Ramadanov, and Hassan Tarek Hakam. Analysis and statistics were performed by Robert Hable and Robert Prill. The first draft of the manuscript was written by Mikhail Salzmann and Nikolai Ramadanov. All authors commented on previous versions of the manuscript. All authors read and approved the final manuscript.

## CONFLICT OF INTEREST STATEMENT

The authors declare no conflict of interest.

## ETHICS STATEMENT

Ethical approval is not applicable for systematic reviews.

## Supporting information

Supporting information.

Supporting information.

Supporting information.

## Data Availability

The data are available from the corresponding author on request.
