# Supplementary material for: Artificial intelligence‐based assessment of leg axis parameters shows excellent agreement with human raters: A systematic review and meta‐analysis
Source: Knee Surg Sports Traumatol Arthrosc. 2024 Jul 21;33(1):177–90. doi: 10.1002/ksa.12362 (PMC11716349; doi:10.1002/ksa.12362)
Supplement: Supplementary file 2 — Supporting information. [file KSA-33-177-s001.docx]

Table: Tabular summary of the aim, population and intervention of the included studies:

| Study | Aim | Population / Radiographs assessed | Intervention / AI Evaluation Software |
| --- | --- | --- | --- |
| Archer et al. (2022) | 1.Assessment the reliability of a **previously developed** AI-based image recognition software as compared to muli-reader evaluation.  2.Assess the time saved using the software and inter-reader assessment. | Lower leg radiographs of 85 (132 radiographs) patients:   1. 49 females and 36 males between the ages of between the ages of 18 and 100. 2. Diagnosis was based on a consensus of two radiologists. 3. Measured angles included: 4. *HKA* 5. *aTFA* 6. *JLCA* 7. *mLDFA* 8. *mMPTA* 9. *mLDTA* 10. *LLD*   *Hence, the goal was angle measurement and not the establishment a diagnosis of hip dysplasia.* | Deep-learning-based software IB Lab LAMA; ImageBiopsy Lab, Austria. |
| Erne et al. (2022) | 1.Development and Validation of a **Novel Algorithm** for the assessment of lower-limb alignment angles.  2.Comparision of the generated measurements to human expert measurements. | Radiographic images of 119 patients who have undergone **total knee arthroplasty.**   1. 46 Males and 73 Females between the ages of 44 and 85 (Mean age 66). 2. Full length weight bearing radiographs of 3 months preoperatively and 3 months postoperatively were included. 3. Measured angles included: 4. mFAmTA 5. FSAmTA 6. mMPTA 7. mLDFA 8. mLDTA   *This study analyzed knee angles of retrospectively obtained radiographs where patients with a minimal time elapse of 3 months after total knee arthroplasty.* | Fully automated AI based algorithm containing 5 deep CNNs created by the authors of the study.  Storage of the image in DICOM.  Workflow: data input and training the artificial intelligence is described in the study: Image input, segmentation, landmark placement, projection, and parameter visualization. |
| Jo et al. (2022) | 1.Conception and validation of a **novel algorithm** capable of recognizing anatomical landmark and measuring angles in FLR. | 11212 FLRs were obtained from a single center.   1. 8275 Females and 2937 Males with and average age of 62.7 (STD 13) was obtained. 2. 15 anatomical landmarks were marked and trained for including: centre of the femoral head, medial and lateral distal points of the femur, intercondylar fossa, medial and lateral tibial articular wedges, medial and lateral spines of the tibia, intercondylar eminence, midpoints of the medial and lateral tibial plateau, medial and lateral edges of the talar dome, centre of the talar dome, and the tip of the fibular head. 3. Four anatomical angles were calculated: 4. mLDFA 5. MPTA 6. JLCA 7. HKAA   *In other words, the study aimed at creating and testing a newly formed AI-recognition software.* | The software included total of 4 CNNs were used to identify anatomical landmarks:   1. Cropping and resizing of the FLR; 2. CNN 1: identifies the hip, knee, and ankle joints. 3. Extraction of cropped recognized joints. 4. CNN 2: identification of anatomical landmarks of the hip. 5. CNN 3: identification of anatomical landmarks of the knee 6. CNN 4: identification of anatomical landmarks of the ankle. 7. Measurement of angles between identified anatomical landmarks. |
| Larson et al. (2022) | 1.Conception and validation of a **novel algorithm** capable of generating radiologic reports including length, angles, hardware description and laterality differences.  2.Inclusion of a surgical hardware detection system to report post-surgical changes and convey limitations. | 643 images were used to train the AI software. 220 images were left out to be analyzed. All images were retrospectively analyzed at a single center.   1. Specific demographic information was not presented. 2. The following anatomical landmarks were used to train the AI: femoral head apex point, center of the femoral head, ischium point, intercondylar notch point, tibial spine point and the ankle mortise point. 3. Two angles were calculated: 4. The mechanical angle 5. The pelvic tilt angle.   Other measurements included total leg, femoral and tibial lengths. | To generate the final report, the AI-based software handles theimported images followingly:   1. Landmark detection by Faster-RCNN101. 2. Prediction preprocessing. 3. Measurement calculation. 4. Hardware detection 5. Report generation |
| Mitterer et al. (2023) | 1.The aim of the study was to evaluate a **previously developed** commercially available software.  2.Outcomes of interest included measured angles and lengths. | 110 weight-bearing LLR were evaluated:   1. The participants included 62 males and 40 females with a mean age of 40.8 (STD 11.8) 2. Some 8 patients had radiographs of both legs 3. 55 images were radiographs of DFO patients 4. 55 images were radiographs of HTO patients   *The study aimed at evaluating a previously developed software and hence no landmarks were identified.* | IB Lab LAMA (Leg Angle Measurement Assistant 1.13.16, ImageBiopsy Lab, Vienna, Austria.   1. The algorithm was trained using 15000 LLRs from the OAI, MOST, CHECK and five Austrian websites. 2. In case a landmark cannot be identified, outputs are suppressed. |
| Moon et al. (2023) | 1.Development of a deep learning-based **novel algorithm** to quantify the lower extremity alignment.  2.Assement of the performance and reliability of the software. | 450 LLRs were obtained from 450 patients.   1. LLRs with artificial joints or skeletal or fibrous dysplasia were excluded (originally 770 LLRs). 2. 200 LLRs were used to train the AI. 50 LLRs were used to validate the AI. 200 were used to test the AI.   Measured angles included:   1. mLPFA 2. mLDFA 3. mMPTA 4. mLDTA 5. MAD 6. mJLCA 7. mTFA 8. aMPFA 9. aLDFA 10. NSA 11. aMPTA 12. aLDFA 13. aTFA   Additionally, full leg length, femoral length and tibial length were measure for each radiograph. | To generate final measurements the algorithm had the following steps to process:  Conception:   1. Detection of ROIs on the femur, tibia, and ankle. 2. Cropping and semantic segmentation then extraction of mask images. 3. Extracted images used to measure parameters. Detected landmarks used to calculate angles and lengths.   Training:  YOLOv5 used to identify ROIs. HarDNet-MSEG was used to create images for each ROI. CLAHE to enhance low contrast images. |
| Pei et al. (2021) | 1.Development of a **Novel Algorithm** for the assessment of deformities of lower limb radiographs.  2.Assessment of the accuracy of this developed AI model. | 676 images were extracted for the purposes of this study.   1. 112 males, 286 females aged 5 to 85 from taken between October 2018 and august 2020. 2. 80% were used to train the AI model- 3. 20% of the images were used to validate the then developed model. 4. The only measured ankle was the HKA. 5. Exclusion criteria are as the following:   (a) hip replacement;  (b) severe developmental dysplasia of hip; (c) knee replacement;  (d) artificial limb;  (e) poor quality images. | A U-Net system was used to evaluate the radiographs.   1. Encoding part: made up of a convolution layer. 2. Decoding part: made up of a deconvolution layer.   The centers of the following joints were marked to allow for the measurement of the HKA angles: Femoral head, knee and ankle. |
| Schock et al. (2021) | 1.**Development** of an AI-based automated quantitative analysis software for lower leg radiographs.  2.**Validation** of the AI model. | 225 LLRs were obtained between January and September of 208.   1. 157 males and 78 females with and average age of 26 (23 standard deviation) 2. 109 LLRs were used to train the CNN 3. 40 LLRs were used for validation. 4. 106 were used to subsequently test the generated AI software. 5. Angles included: 6. HKA 7. AMA | A U-Net model (a CNN based model) with a segmentation-based algorithm similar to the previously described AI-models was developed and later validated.  Detected landmarks:   1. Center of femoral head, 2. Femoral intercondylar point (apex of femoral notch), 3. Tibial interspinous point (midpoint of the tibial spines), 4. Tibial midplafond point (midpoint of the outer edges of the malleoli along the tibial plafond). |
| Schwarz et al. (2022) | Assessment of a previously developed software that assesses measurements of LLRs in the postoperative phase following TKA. | 1312 LLRs with postoperative knees following **total knee arthroplasties** were assessed.   1. 845 females and 434 males with a mean age of 69 (9 years standard deviation) 2. Exclusion criteria were 3. unicondylar knee replacements 4. incorrect positioning 5. poor visibility 6. missing calibration balls 7. abnormal cropping. 8. Angles included: 9. HKA 10. FCA 11. TCA | The IB Lab LAMA software version 1.03, IB Lab GmbH, Vienna, Austria was used. This AI-based software was trained using 1500 radiographs from the OAI database. |
| Simon et al. (2021) | Assessment of a previously developed software for LLR assessment. | 295 LLRs from 284 patients.   1. 287 female and 97 male 2. Exclusion criteria: 3. Artifacts 4. Poor visibility 5. Incorrect positioning 6. Abnormal cropping 7. Missing Calibration ball 8. Metal implants 9. Angles: 10. HKA 11. AMA 12. JLCA 13. mLDTA 14. mLPFA 15. mMPTA 16. MAD 17. Leg, femoral and tibial lengths | U-Net based LAMA Software Version 1.03.17, IB Lab GmbH, Vienna Austria.  Training was conducted on 15000 radiographs from OARSI, MOST and CHECK |
| Steele et al. (2023) | 1.Development of an AI model that accurately measures lower limb alignment  2.Validation of that software  3.Evaluate the distribution between sexes  4.Evaluate the differences based on the Kellgren-Lawrence grade | Radiographs from 4796 patients between the ages of 45 to 79 (average 61 years) obtained between 2004 and 2015 were evaluated.   1. Patients with knee flexion contracture or hyperextension were excluded from the study. 2. Measured Angles: 3. LFDA 4. MPTA 5. HKA 6. JLO 7. Knee phenotypes based upon CPAK classification was used to group the radiographs in 3 categories. | U-NET convolutional neural network to identify bony landmarks, measure angles and classify along the CPAK classification.  250 images were used for training and validation. |
| Stotter et al. (2023) | Assessment of a previously developed software that evaluates LLRs | LLRs of 95 patients pre- and postoperatively following **high tibial osteotomy**.   1. 41 Female and 54 Male 2. Mean age: 46.9 +/- 7.6 years 3. Measured angles included: 4. HKA 5. MAD 6. JLCA 7. MPTA 8. mLDFA | U-Net based LAMA software version 1.04.15, CE version, Image Biopsy Lab, Vienna, Austria.  Previously trained on 15 000 radiographs obtained from the OAI, MOST and CHECK. |
| Tsai (2022) | 1.Development of a **novel AI-based algorithm** for the assessment of lower limb alignment in children. | 575 LLRs of children below the age of 18 years were obtained between July and September of 2019.   1. No specific exclusion criteria were used to provide the most comprehensive possible search. 2. Only the HKA angle was calculated | A CNN Pyro4.79, Uber AI labs (pyro.ai) that directly regresses across special coordinates (specific landmarks) was used.  To reduce the memory requirement and the training time, images were cropped down. |

mPTA: Angle between the mechanical axis of the tibia and the tibial plateau knee joint line measured medially; mLDFA: angle between the femoral mechanical axis and the femoral joint line measured laterally. mLDTA: angle between the tibial mechanical axis and the tibial plafond measured laterally; CNN: convoluted neural network; DICOM: digital imaging and communications in medicine; OAI: osteoarthritis initiative ; HKA: hip knee angle; MOST: Multicenter Osteoarthritis Study; CHECK: Cohort Hip and Cohort Knee study; DFO: distal femoral osteotomy; HTO: high tibial osteotomy; ROI: region of interest; MAD: mechanical axis deviation; mJLCA: joint line convergence angle; mTFA: mechanical tibiofemoral angle; aMPFA: anatomical medial proximal femoral angle; aLDFA: anatomic lateral distal femoral angle; NSA: neck shaft angle; aMPTA: anatomical medial proximal tibial angle; aLDFA: anatomic lateral distal femoral angle; aTFA: Anatomical tibifemoral angle
