# Supplementary material for: Artificial intelligence‐based assessment of leg axis parameters shows excellent agreement with human raters: A systematic review and meta‐analysis
Source: Knee Surg Sports Traumatol Arthrosc. 2024 Jul 21;33(1):177–90. doi: 10.1002/ksa.12362 (PMC11716349; doi:10.1002/ksa.12362)
Supplement: Supplementary file 3 — Supporting information. [file KSA-33-177-s003.docx]

| Study | Comparators | Outcomes | Recommendations and Limitations |
| --- | --- | --- | --- |
| Archer et al. (2020) | Two radiologist with unclear levels of training. | Primary outcome:  AI-based evaluation of the before-mentioned angles was successful.  Secondary Outcome:  The AI-based software performed the reading quicker. 91% and 93.9 % quicker than readers 1 and 2. | Recommendations:   1. Clinical: 2. Deep learning-based software have the potential to accurately measure hip angles. 3. These algorithms have the potential to perform readings quicker and are financially more rentable than manual reading. 4. Limitations: 5. Input was limited due to the selection of higher quality images. 6. 11 exclusions due to the incorrect placement of the anatomic landmarks. |
| Erne et al. (2022) | 2 orthopaedic surgeons measured the angles using a radiologic software suit: MediCAD 2D classic; mediCAD Hectec GmbH, Altdorf/Landshut, Germany.  *Details regarding the background of the mentioned orthopaedic surgeons such as background and years of experience was not given* | Primary outcome:  Conception of an artificial intelligence-based software for knee angle measurement in full length radiographs of the lower limb.  Preoperative Radiographs:   1. mMPTA, mLDFA and FSAmTA could be detected in 98.9% of cases. 2. mLDFA and mFAmTA could be detected in 92.6% of cases.   Postoperative Radiographs:   1. mMPTA and mLDTA could be detected in 97.1% of cases. 2. FSAmTA, mLDFA and mFAmTA detection rates were 95.2%, 94.3% and 92,4% respectively. | Recommendations:   1. Clinical:   The use of AI-based knee angle measurements is recommended by the authors as it is a timesaving, fatigue-free and objective examination procedure that allows to process a large volume of knee radiographs in a short amount of time.   1. Scientific:   a. Extension of the current available algorithms might the next best step.  b. Combination of several algorithms of different research can create a more comprehensive program.  Limitations:   1. Fundamental procedural deficiencies due to inherent techniques when FLR radiographs are performed. 2. Size of validation dataset was limited. 3. Only TKR patients were included although FLR are obtained for a variety of conditions. |
| Jo et al. (2023) | One orthopedic specialist with 14 years of experience, 2 orthopedic surgeons and 2 medical students.  *The difference between orthopedic surgeon and orthopedic specialist as well as the experience and subspecialty areas of the orthopedic personnel remains rather unclear. Furthermore, it is unclear how medical students were trained to be able to evaluate the radiographic images.* | Primary outcome:  Conception of an AI-based image recognition and evaluation software able to provide angle measurements.  Secondary outcome:  Determination of the inter-observer reliability in the human group as well as the intra-group observer reliability between AI and humans. | Recommendations:   1. Clinical: 2. AI-based landmark recognition provides superior angle measurement results for FLR. 3. The software can be reliably used for clinical applications especially in patients with indications to total knee arthroplasty, distal femoral osteotomy, and high tibial osteotomy. 4. Scientific:   Using joint classifiers and anatomical landmark recognition are the best respective steps to ensure an accurate AI-based angle measurement.  Limitations:  The deep learning software had difficulties identifying the medial and lateral wedges of the tibial plateau when osteophytes were present. |
| Larson et al. (2022) | 3 fellowship trained musculoskeletal radiologists. | Primary Outcome:  Generation of a new software that enables angle measurements in LLRs.  Secondary Outcome:  Excellent reliability between AI generated and human measurements of angles in LLRs. | Limitations:  The main limitation of this study was the limited dataset used to train the AI. |
| Mitterer et al. (2023) | 3 Human readers using the mediCAD v6.0; Hectec GmbH, Landshut, Germany.  *The readers and their level of training were not further described in the study.* | Primary outcome:  Good to excellent correlation is described by the authors of the study.  Secondary Outcome:  The AI-based reading was significantly faster than manual readings.  *Most faults were due to inaccurate identification of tibial joint lines.* | Recommendations:  None.  Limitations:   1. The presence of hardware challenged the AI-based measurement of angles. 2. Positioning and rotational deviation can lead to significant errors of measurements of parameter. 3. The wide range of patients could have led to heterogenous anatomical characteristics. 4. Retrospectively, errors in AI-applications can not be detected due to the “black box phenomenon”. |
| Moon et al. (2023) | One board certified radiologist with 18 years of experience. | Primary Outcome:  Generation of a new software that enables angle measurements in LLRs.  Secondary Outcome:  Excellent reliability between AI generated and human measurements of angles in LLRs. | None mentioned. |
| Pei et al. (2021) | Three orthopaedic surgeons with 13, 10 and 7 years of experience. | Primary outcome:  Conception of an AI-based image recognition and evaluation software able to provide HKA measurements.  Secondary outcome:  Excellent interrater correlation. The agreement between all orthopedic surgeons was very high for a given radiograph. | No specific recommendations. |
| Schock et al. (2021) | Two clinical radiologists with 3 years of experience were used as comparators. | Primary outcome:  Conception of an AI-based image recognition and evaluation software able to provide HKA and AMA measurements.  Secondary outcome:  Excellent interrater correlation. The agreement between the two clinical radiologistswas very high for a given radiograph. | Clinical recommendations:  Automatic quantification of AI-generated software still needs to be double-checked by clinical radiologists.  Limitations:   1. Data diversity was limited by the use of single leg radiographs. 2. The training data was tailored to a younger population thereby limiting the diversity of the dataset used for training. 3. No other matrices then those previously mentioned were used to assess lower leg malalignment. 4. The test-retest reliability was not assessed |
| Schwarz et al. (2022) | Two orthopedic surgeons who routinely performed LLR measurements (no further detailed information) annotated the angles usind mediCAD, Hectec GmbH, Landshut, Germany. | Primary outcome:  Excellent correlation between manual reads and AI measured angles.  Secondary outcome:  In 92.1% of cases the investigated implants were correctly detected. | No specific recommendations. |
| Simon et al. (2021) | Two orthopedic surgeons that routinely perform LLRs with 3 years of experience. | Primary Outcome:  Excellent reliability for angle and length measurements. | Scientific:   1. As small differences in landmark setting can result in significant differences between readings, human and AI generated measures cannot always be contrasted.   Clinical:   1. Enables radiologists and surgeons to save time and manage workloads efficiently.   Limitations:   1. Only two observers with the same level of expertise were included in this study. |
| Steele et al. (2023) | One fellowship trained surgeon. (no further information was provided). | Primary outcome:  Development of a new AI-based knee angle measurement software.  Secondary outcomes:  CPAK classification was like the originally described work.  Women had a significantly higher rate of varus alignment.  No significant distribution regarding | Limitations:   1. Exclusion of images where landmark detection failed. 2. LLRs showing a flexion contracture of more than 10 degrees were excluded from the analysis due to the determination to eliminate poor AP views. |
| Stotter et al. (2023) | Three investigators: 2 orthopedic surgeons and 1 musculoskeletal radiologist.  All had a minimum of 5 years in experience with musceloskeletal imaging. | Primary outcome:  AI-based automated long leg alignment measurements produce reliable results. | Recommendations:   1. Clinical: AI-based measurements of angles can be integrated into clinical practice, especially when human resources are scarce. 2. Further adjustment can be made whilst training the AI to optimize reading.   Limitations:   1. Small sample size 2. Dependance of measurements on the quality of LLRs 3. Human measurements might not be infallible and have some errors. |
| Tsai et al. (2022) | Ground truth was established by a pediatric musculoskeletal radiologist. | Primary outcome:  Development of an AI-based algorithm for HKA assessment in children. The ground truth was established by a specialized pediatric musculoskeletal radiologist | Clinical:  This CNN has the potential to reduce the time used to evaluate the HKA in LLRs of children.  Limitations:   1. Retrospective methodology. 2. Reduced resolution of input radiographs. 3. Systematic bias die to the evaluation by only one radiologist. |
